# Pirenperone relieves the symptoms of fragile X syndrome in Fmr1 knockout mice

**DOI:** 10.1038/s41598-022-25582-8

**Published:** 2022-12-05

**Authors:** Yujeong Kim, Se Jin Jeon, Edson Luck Gonzales, Dongpil Shin, Chilly Gay Remonde, TaeJin Ahn, Chan Young Shin

**Affiliations:** 1 Department of Pharmacology and Department of Advanced Translational Medicine, School of Medicine, Konkuk University, Seoul, 05029 Republic of Korea; 2 Department of Integrative Biotechnology, College of Science and Technology, Sahmyook University, Seoul, 01795 Republic of Korea; 3 Department of Life Science, Handong Global University, Nehemiah 36, Handong-ro 558, Pohang, 37554 Republic of Korea

**Keywords:** Drug discovery, Neuroscience

## Abstract

Fragile X syndrome (FXS) is a neurodevelopmental disorder that is caused by the loss of Fragile X-linked mental retardation protein (FMRP), an RNA binding protein that can bind and recognize different RNA structures and regulate the target mRNAs’ translation involved in neuronal synaptic plasticity. Perturbations of this gene expression network have been related to abnormal behavioral symptoms such as hyperactivity, and impulsivity. Considering the roles of FMRP in the modulation of mRNA translation, we investigated the differentially expressed genes which might be targeted to revert to normal and ameliorate behavioral symptoms. Gene expression data was analyzed and used the connectivity map (CMap) to understand the changes in gene expression in FXS and predict the effective drug candidates. We analyzed the GSE7329 dataset that had 15 control and 8 FXS patients’ lymphoblastoid samples. Among 924 genes, 42 genes were selected as signatures for CMap analysis, and 24 associated drugs were found. Pirenperone was selected as a potential drug candidate for FXS for its possible antipsychotic effect. Treatment of pirenperone increased the expression level of Fmr1 gene. Moreover, pirenperone rescued the behavioral deficits in Fmr1 KO mice including hyperactivity, spatial memory, and impulsivity. These results suggest that pirenperone is a new drug candidate for FXS, which should be verified in future studies.

## Introduction

Fragile X syndrome (FXS) is an X chromosome-related genetic disorder that is associated with neurodevelopmental problems such as hyperactivity, intellectual disability, social problems, and impulsive behaviors. FXS is caused by a mutation of the fragile X mental retardation 1 gene (*FMR1*) located at Xq27.3. This mutation initiates an abnormal repetitive expansion of the trinucleotide CGG in the 5′ untranslated region of the FMR1 gene. This causes silencing of the FMR1 gene and results in the deficiency of its genetic product, FMRP^1^. Because FMRP contributes to the synaptogenesis and maintenance of synaptic function, brain development, and neural plasticity, lack of FMRP will cause neural mRNA alternations, increases neural protein synthesis, and imbalance in the excitatory/inhibitory synaptic transmission, which is one of the major phenotypes in FXS^2^. Several reports suggested a causal relationship between the level of FMRP and the clinical characteristics of patients with FXS. The emotional and cognitive phenotypes of FXS patients depend on the degree of methylation for FMR1 and its number of repeats. This means that the lower the level of FMRP, the more severe the symptoms will be^2^. Common physical features of FXS include a long face, macroorchidism, prominent ears and jaws, flat feet, and joint hypermobility. FXS patients have developmental problems such as mental retardation, intellectual disability, and language delay^2^. About 15% of patients have seizures during their childhood^3^ and about 20% have strabismus^4^.

Pharmacological and educational treatments for the management of behavioral and medical problems such as attention deficit hyperactivity disorder (ADHD)-like symptoms, aggressive behaviors, anxiety, and hyperarousal of FXS patients were performed^4^. In previous clinical studies, a positive response to treatment was defined as the type of improvement in the behavioral symptoms for 6 months with no major side effects leading to discontinuance of the medication. The success rate of each medication was 53% for selective serotonin reuptake inhibitor (SSRI), 62% for alpha-adrenergic agonists, and 54% for antipsychotics. In addition, SSRIs are one of the most used forms of treatment for adults or females with FXS. This helped alleviate social anxiety and withdrawals, especially in females. Alpha-adrenergic agonists were effective in treating hyperactivity and over-arousal as well as in alleviating sleep problems in young children with FXS. The antipsychotics helped target the aggressive behavior as well as the irritability associated with FXS^5^. Abnormal signaling pathways activated by metabotropic glutamate receptor (mGluR) are considered plausible treatment targets for FXS^6^. Unfortunately, clinical trials using these approaches were not successful in requesting other target molecules to test for the treatment of the detrimental condition. The reduction of FMRP regulated the expression of individual proteins as well as their activity. Among these, increased expression of α-amino-3-hydroxy-5-methyl-4-isoxazole propionic acid (AMPA) receptors and regulation of activity of receptors or proteins that modulate glutamate signaling has been repeatedly suggested as a potential mechanism to treat FXS. MMP9, MAP1B, PSD95, CaMKII, Arc, STEP, PIKE, APP, potassium channel Kv 3.1b, and others are also among the synaptic proteins that correspond to the FMRP target mRNAs and elevated in the fmr1 knockout (KO) mice^2^. As an RNA binding protein, FMRP can bind and regulate its target mRNAs’ expression which results in the abnormal gene expression signature in FXS compared to normal control. Considering the importance of the level of FMRP protein in the modulation of clinical phenotypes of FXS and its role as a modulator of translation by binding to target mRNAs, we first hypothesized that drugs that have the potential to normalize the altered gene expression profile in fmr1 KO mice or FXS patients may alleviate the neurobehavioral deficits seen in fmr1 KO mice. These drugs are expected to target the brain and modulate aberrant neural activity, especially preferring molecules that modulate neural transmission. Based on this hypothesis and assumption, we identified a candidate molecule and further examined its therapeutic effects in the fmr1 KO mice model of FXS. Along with this hypothesis, recently, transcriptomic data have been analyzed to investigate disease-drug and disease-disease relationships^7^ in the drug discovery process. Transcriptomic data is composed of genome-wide gene expression profiles and it gives experimenters information about specific cells, tissues, or organisms' transcript levels under the experimental conditions^8^. The gene expression omnibus (GEO), which is administered by the National Center for Biotechnology Information (NCBI), is widely used to get functional genomic data publicly. By analyzing transcriptomics data, it is easier to understand the network of differentially expressed genes (DEG) from specific biological processes, diseases, or drugs. With regards to drug development, using DEG analysis can discover potential targets for disease therapeutics^8^. Connectivity map (CMap) is a method and database that helps to find molecules that induce or revert the gene expression signature of interest. CMap database has over 1.5 million gene expression profiles with around 5000 small molecule compounds-induced transcriptomic data sets. CMap utilizes a gene expression signature as an input and calculates the enrichment score. If a drug candidate molecule reverts the gene expression signature the score is negative. A positive score means a drug candidate molecule induces the gene signature. CMap output provides a list of candidate molecules' enrichment scores along with their statistical significance. CMap has been widely used to repurpose drugs, and discover new lead compounds in various diseases such as hair growth^9^, acute myelogenous leukemia^2^, smoking cessation^2^, cancers^2^.

As a new candidate for FXS treatment, pirenperone was further validated for its potential therapeutic effects both in vitro and in vivo. This approach and study may offer chances for novel therapeutic development against FXS and can be expanded to other CNS disorders that also need effective treatment.

## Results

### DEGs through transcriptome analysis

**Figure 1:**
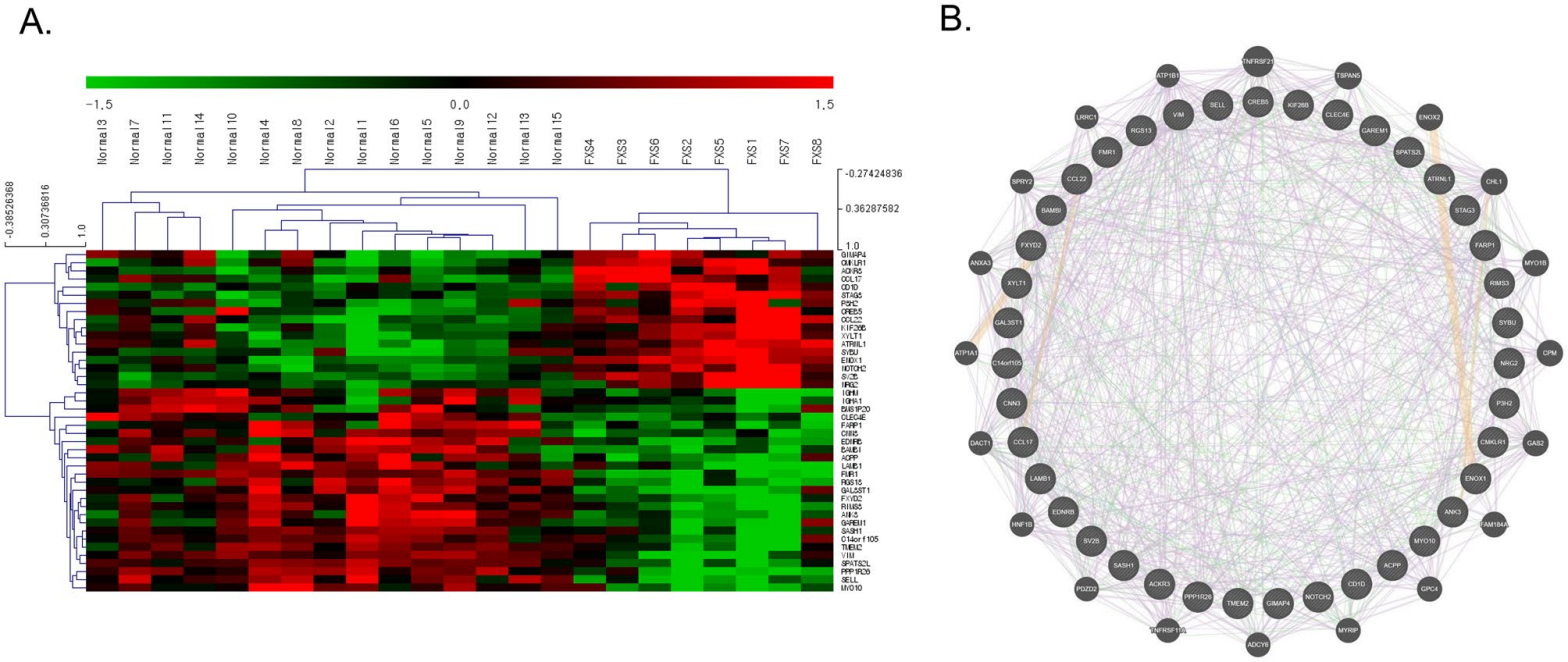
42 genes were selected as DEGs between FXS and healthy control from GSE7329 data. (**A**) Heatmap of DEGs. (**B**) Molecular relationship between DEGs and associated genes. DEGs are located at the inner circle, associated are at outer. The color of the edge represents co-expression (purple), shared protein domain (grey), genetic interaction (green), predicted association (orange).

To make signatures for CMap analysis, representative FXS transcriptome data were analyzed (GSE7329). Lists of up-and down-regulated genes based on the 90th percentile value of mean difference calculation are shown as a heatmap (Fig. 1A). There were 17 genes for up-regulated and 25 genes for down-regulated. Some of the DEGs (e.g. *ATRNL1*^2^, *FMR1*^2^, *LAMB1*^2^) were previously reported as issued genes related to neurodevelopmental diseases. DEGs were further analyzed by GeneMANIA to reveal the molecular functional relationship. Two genes in the subnetwork, *GAREM1*, and *ACKR3*, along with co-expressing genes of *SPRY2* and *TNFRSF11A* are revealed to be involved in the positive regulation of ERK1 and ERK2 cascade function (Fig. 1B). Interestingly, it has been reported that abnormal ERK activation in tissues from Fmr1 KO mice and blood lymphocytes from FXS patients^2^. However, no significant functional enrichment was found with 42 DEGs when FDR is controlled at 0.2. The whole DEG list is uploaded as Supplement material (Supplementary Table S1).

### Mapping DEGs to CMAP

BY applying DEGs to CMap, 56 drugs with *p* values lower than 0.05 were deducted and among them, 25 drugs with a negative enrichment score to change the level of DEG oppositely were selected (Table 1). Among 25 drugs, 11 drugs are approved drugs by the US FDA. Pirenperone, a 5-hydroxytryptamine receptor (HT) 2A antagonist, was selected as a potential candidate for FXS because of its antipsychotic effects^2^. It had a − 0.711 enrichment value with a *p* value of 0.00434 from the CMap analysis.

**Table 1:** Top chemicals correlated with up and down signatures in the connectivity map by using DEGs.

CMap name	Mean	n	Enrichment	*p*	Specificity	Percent non-null
Thiamine	− 0.727	3	− 0.96	0.00018	0	100
SC-560	− 0.58	3	− 0.899	0.00194	0.0121	100
Hycanthone	− 0.312	4	− 0.803	0.00294	0.0168	50
Biperiden	− 0.355	5	− 0.723	0.00348	0.1088	60
Myosmine	− 0.38	6	− 0.665	0.00399	0.0111	83
Pirenperone	− 0.415	5	− 0.711	0.00433	0	80
MK-886	− 0.716	2	− 0.936	0.00861	0	100
Bacitracin	− 0.582	3	− 0.837	0.00861	0.0242	100
Vinburnine	− 0.413	4	− 0.742	0.00871	0	75
Prestwick-983	− 0.621	3	− 0.834	0.00915	0.0206	100
Iohexol	− 0.416	4	− 0.724	0.0118	0.0511	75
Chloropyrazine	− 0.515	4	− 0.723	0.012	0.0195	75
Betazole	− 0.424	5	− 0.628	0.01846	0.007	80
Rilmenidine	− 0.393	4	− 0.694	0.01866	0.0274	75
Fenofibrate	− 0.412	3	− 0.789	0.01913	0.0581	66
Eticlopride	− 0.282	4	− 0.69	0.02003	0.0379	50
Oxolamine	− 0.222	4	− 0.689	0.02029	0.0461	50
Lisuride	− 0.258	5	− 0.614	0.02363	0.2869	60
Prestwick-857	− 0.341	4	− 0.673	0.02564	0.051	75
Chenodeoxycholic acid	− 0.444	4	− 0.673	0.02564	0.1308	75
Bretylium tosilate	− 0.3	4	− 0.671	0.02616	0.0385	50
Lovastatin	− 0.287	4	− 0.667	0.02765	0.0388	50
Quipazine	− 0.237	4	− 0.648	0.03648	0.0315	50
Clidinium bromide	− 0.491	4	− 0.638	0.04116	0.0245	75
Harmol	− 0.433	4	− 0.63	0.04603	0.1585	75

### Pirenperone treatment reversed the expression signature of the Fmr1 gene in control rat cortical neuron

**Figure 2:**
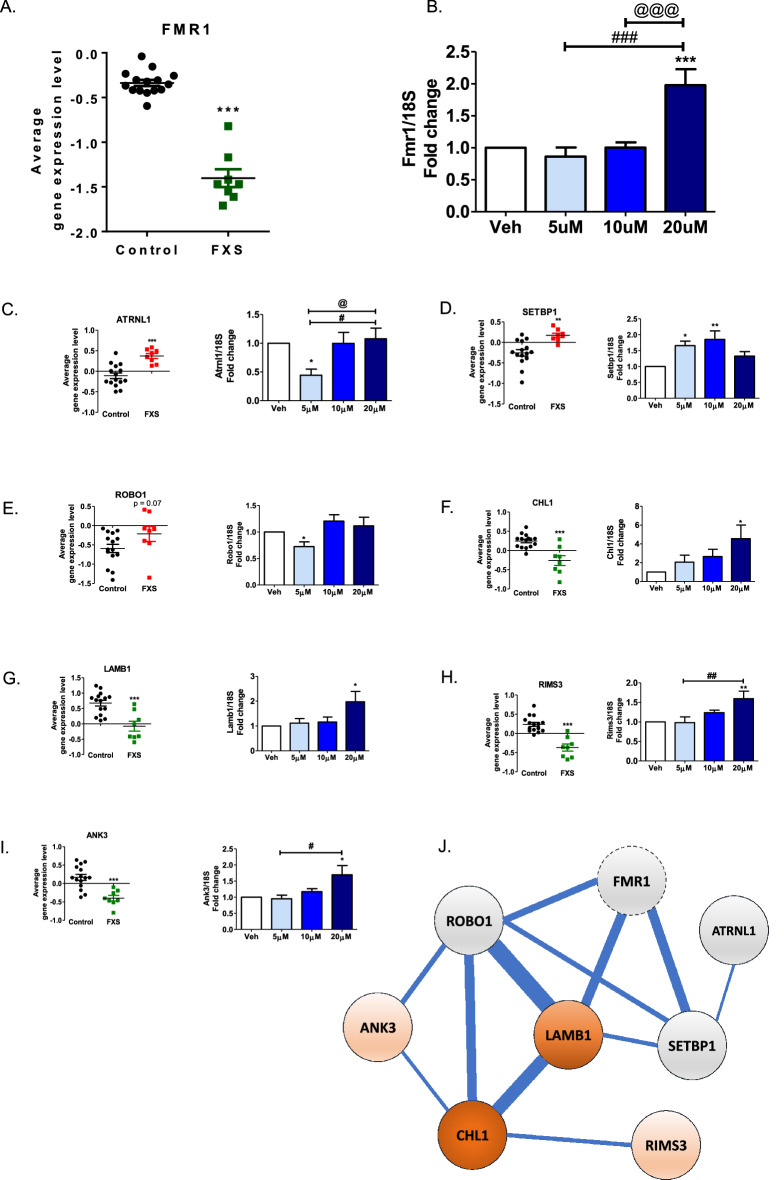
Upregulation of Fmr1 gene expression upon pirenperone treatment (**A**) *FMR1* gene expression was significantly down-regulated in human FXS patients. Unpaired t-test. Control versus FXS, ***p < 0.001 (**B**) Fmr1 gene expression level was significantly up-regulated after pirenperone treatment at a dose of 20 μM in cortical neuron. (**C**) Gene expression level for *ATRNL1* in human patients (left) and real-time PCR result in cultures (right). (**D**) Gene expression level for *SETBP1* in human patients (left) and real-time PCR result in cultures (right). (**E**) Gene expression level for *ROBO11* in human patients (left) and real-time PCR result in cultures (right). (**F**) Gene expression level for *CHL1* in human patients (left) and real-time PCR result in cultures (right). (**G**) Gene expression level for *LAMB1* in human patients (left) and real-time PCR result in cultures (right). (**H**) Gene expression level for *RIMS3* in human patients (left) and real-time PCR result in cultures (right). (**I**) Gene expression level for *ANK3* in human patients (left) and real-time PCR result in cultures (right). One-way ANOVA, post hoc test: Bonferroni’s multiple comparison test, Vehicle versus 20 μM, ***5 μM versus 20 μM, ^###^10 μM versus 20 μM, ^@@@^*p* < 0.001, number of samples per group = 12. (**J**) Gene expression change after treatment of pirenperone. Color in the circle represents expression difference between the pirenperone and vehicle treatment (red: higher expression in pirenperone, grey means no difference). The thickness of edges represents the number of supporting evidence of a co-expressional relationship between genes.

To validate the findings from the transcriptomic analysis, the expression level of the Fmr1 gene, one of the genes which regulate FMRP, was confirmed with qRT-PCR by treating pirenperone in the control rat cortical neuron cell (Fig. 2A,B). Other input genes were also used to confirm the reversal of signature in our study: four genes were downregulated (*LAMB1*, *ANK3*, *RIMS3*, *and CHL1*) and three genes were upregulated (*ROBO1*, *SETBP1*, and *ATRNL1*) in FXS. Pirenperone treatment at 20 μM elevates gene expression of originally downregulated genes in FXS (4.56, 2.04, 1.595, and 1.732 times at *LAMB1*, *CHL1*, *ANK3*, and *RIMS3*, respectively), where it marginally changes the gene expression of originally upregulated genes (Fig. 2C–I). The results suggest that the expression levels of genes in cortical neurons which were mutated and causative for FXS were counter-regulated by pirenperone. The co-expression gene network of seven genes are functionally enriched in epithelial cell proliferation (FDR q-value: 0.032), basement membrane (q-value: 0.09), extracellular matrix disassembly (q-value: 0.12) (Fig. 2J). Our findings in conjunction with the known mechanism of such inputs may suggest pirenperone possibly rescue FXS symptoms by affecting the synaptic coverage of neurons.

In addition, pirenperone treatment significantly induced the FMRP expression in cortical neuron cultures (*p* value: 0.0152, Fig. 2B) demonstrating that pirenperone may be effective in the recovery of FMRP transcript levels in FXS.

### The hyperactivity of Fmr1 KO mice was rescued by pirenperone treatment

**Figure 3:**
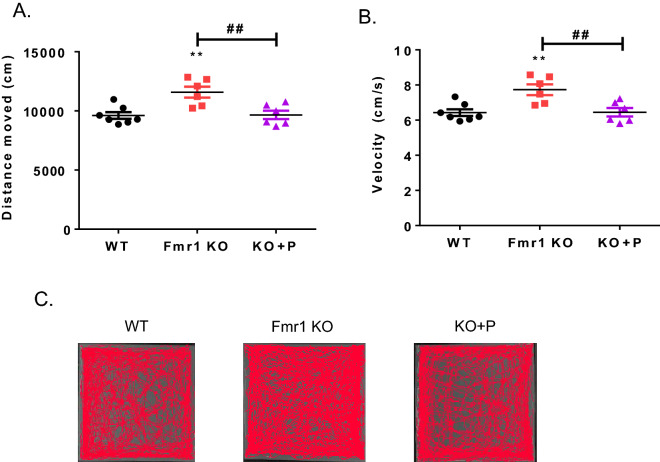
Pirenperone treatment rescued the hyperactivity in the Fmr1 knockout animal. An open field test was performed at the age of 6 weeks. (**A**) After pirenperone treatment, Fmr1 KO mice showed similar distance-moved as WT. One-way ANOVA, post hoc test: Bonferroni’s multiple comparison test, WT versus KO + P (pirenperone), Fmr1 KO versus KO + P, **,^##^*p* < 0.01 (**B**) Pirenperone treatment, normalized higher moving velocity in Fmr1 KO mice to WT level. One-way ANOVA, post hoc test: Bonferroni’s multiple comparison test, WT versus KO + P, Fmr1 KO versus KO + P, **,^##^*p* < 0.01 (Number of samples per group = 6–7) (**C**) Tracking images of animals.

Hyperactivity is one of the most significant features in Fmr1 KO mice^2^. To observe the hyperactivity of Fmr1 KO mice, the open field test was performed (Fig. 3A). Fmr1 KO mice showed hyperactivity with more distance moved and higher velocity than wild-type (WT) animals. After the injection of pirenperone, Fmr1 KO mice with pirenperone treatment showed decreased distance moved and velocity compared to Fmr1 KO mice (Fig. 3B). This result suggests that pirenperone can rescue the hyperactivity phenotype of FXS (Fig. 3C).

### The spatial memory of Fmr1 KO mice was normalized by pirenperone treatment

**Figure 4:**
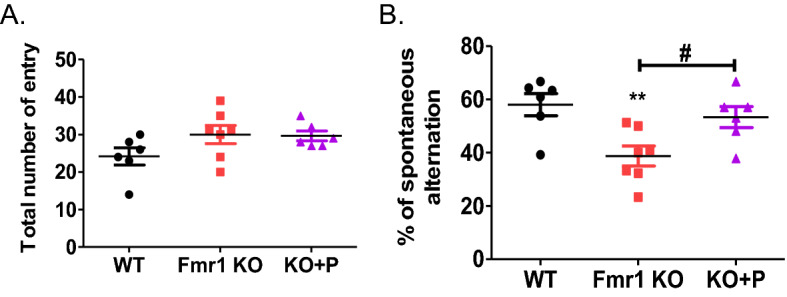
Restored spatial memory and learning ability in Fmr1 knockout mice with pirenperone treatment. Y-maze was performed at the age of 6 weeks by measuring (**A**) total number entry for 8 min recording. (**B**) percent of spontaneous alternation by calculating [(number of alternation/total number of entry − 2) × 100]. One-way ANOVA, post hoc test: Bonferroni’s multiple comparison test, WT versus Fmr1 KO, **p < 0.01, Fmr1 KO versus KO + P (pirenperone), ^#^*p* < 0.05 (Number of samples per group = 6–7).

Fmr1 KO mouse has deficits in learning and memory^10^. Y-maze was performed to confirm whether Fmr1 KO mice have lower spatial memory than WT mice. After the injection of pirenperone, Fmr1 KO mice with pirenperone treatment showed increased spontaneous alternation which suggests better spatial memory in pirenperone-treated mice than Fmr1 KO mice with saline (Fig. 4B). This result implies that pirenperone may improve spatial memory deficits in FXS. There was no significance in the total number of entries (Fig. 4A).

### The impulsivity behavior of Fmr1 KO mice was normalized by pirenperone treatment

**Figure 5:**
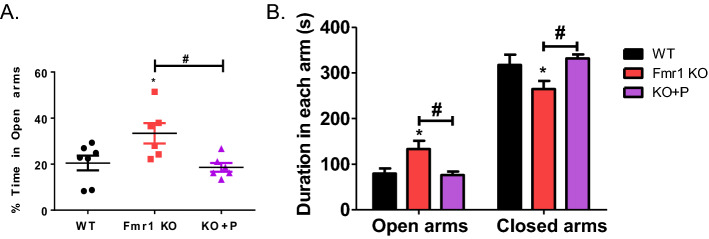
Level of impulsivity was normalized after pirenperone treatment. Elevated plus-maze was performed at the age of 6 weeks. (**A**) The duration of staying at both open and closed arms were measured and the percent time in open arms was calculated using the following formula [(duration in open arms)/(duration in both open and closed arms) × 100]. One-way ANOVA, post hoc test: Bonferroni’s multiple comparison test, WT versus Fmr1 KO, *p < 0.05, Fmr1 KO versus KO + P (pirenperone), ^#^*p* < 0.05. (**B**) The frequency of entering each arm by the group (Number of samples per group = 6–7).

Since impulsivity is a common feature of FXS patients^2^, we examined the impulsive behaviors of Fmr1 KO mice using an elevated plus maze (EPM)^2^. Staying time in the open arm is indicative of impulsivity and anxiety^2^. Fmr1 KO mice stayed more often in the open arms than WT and after pirenperone treatment, the impulsivity-related behavior was normalized to WT level (Fig. 5). The frequency of entering each arm was counted and Fmr1 KO mice entered closed arms more than normal fmr1 knockout mice (Fig. 5). These results suggest that the Fmr1 KO mice revealed higher impulsivity and it is rescued by pirenperone treatment.

## Discussion

In our study, a novel potential drug candidate for FXS was determined through transcriptome analysis. Until recently, one of the most promising drug candidates for the treatment of FXS was group I metabotropic glutamate receptors (mGluR) modulators because mGluRs are regulated by the FMRP and plays important role in the pathophysiology of FXS^2^. Regulation of proteins that are related to glutamate signaling can also be a potential target to treat FXS^2^. Aside from these targets, we here discovered a potential new drug candidate pirenperone to treat FXS by CMap analysis of DEGs, which is helpful to understand the interaction among genes and biological processes^8^.

In our CMap analysis for the new drug candidates, the top 5 chemicals are thiamine, sc-560, hycanthone, biperiden, and myosmine. Among them, thiamine, sc-560, and myosmine were not approved for drugs, and hycanthone, a schistosomicide, targets multidrug resistance protein 1^11^ and is shown to be an effective inhibitor of acetylcholinesterase (AChE) from *Schistosoma mansoni*, but is less potent against AChE from the mammalian origin^12^. Biperiden is a medication used to treat Parkinson's disease and certain drug-induced movement disorders^2^. It relieves muscle rigidity but is not used for neuropsychiatric problems. Thus, we selected pirenperone as a potential candidate for FXS because of its antipsychotic effects^2^. Pirenperone is a selective antagonist of the serotonin (5-hydroxytryptamine, 5-HT) receptor, specifically 5-HT2A^2^. It also has an affinity for 5-HT7^13^ and dopamine receptors but has little effect on 5-HT1 binding^14^. Pirenperone is also reported to be liposoluble which means that it can penetrate the brain easily^15^. The drug is known as an antipsychotic and a tranquilizer that blocks the lysergic acid diethylamide (LSD) effects in animals^16^ implying that has no risk of side effects such as hallucinations.

In this study, the expression of the FXS causative gene *Fmr1* as well as several other ASD-related genes such as *LAMB1*, *CHL1*, *ANK3*, and *RIMS3* was transiently induced by pirenperone treatment in cortical neurons. *LAMB1* gene drives the translation of laminin beta-1 protein, which is an extracellular matrix glycoprotein involved in several processes during embryonic development, such as laminar structure organization in the cerebral cortex, and are known stimulators of neurite outgrowth^17^. Many studies have found the possible involvement of *LAMB1* mutation in the manifestation and severity of ASD^2^. *CHL1* (cell adhesion L1-like) is a gene located in chromosome 3p26.3 that encodes a protein from the L1 family of cell adhesion molecules that are involved in CNS development and synaptic plasticity^18^. Mutations of this gene have been known to impair cognitive function^19^ with possible involvement in the development of ASD^20,21^ or other neurodevelopmental disorders^22^. The *ANK3* gene-encoded AnkyrinG protein is involved in brain development and molecular signaling and is known to play an essential role in intellectual functioning^23^. In addition, the mutation of *ANK3* has been linked to several neuropsychiatric conditions such as ASD^24^, ADHD, bipolar disorder^2^, and schizophrenia^2^, with the involvement of disrupted intellectual function^23^. *RIMS3* is a member of the RIM protein family that serves as an essential component of the presynaptic mechanism for neurotransmitter release and synaptic vesicle fusion^25^. *RIMS3* has been presented as a causal or contributing gene to ASD pathology^2^ and has also been linked to schizophrenia^26^. In this study, several up-regulated genes were not down-regulated in their expression level by pirenperone treatment on cortical neurons in this study, which includes *ROBO1*, *SETBP1*, and *ATRNL1*. While being associated with neuronal differentiation and axon guidance^27,28^, the gene Roundabout homolog 1 precursor (*ROBO-1*) is reported to be differentially expressed in some autistic individuals^2^. *SETBP1* gene encodes for SET binding protein 1^29^ which binds to the SET nuclear oncogene associated with DNA replication^30^. Recurrent de novo missense mutations of this gene are also reported to cause Schinzel-Giedion syndrome, characterized by severe mental retardation, characteristic facial features, and various congenital malformations^29^. A truncating point mutation in *the SETBP1* gene was also identified in a simplex ASD case, as reported by O’Roak and his colleagues^31^. Highly conserved among species, the Attractin-like gene (*ATRNL1*) encodes for a single-pass transmembrane glycoprotein associated with cell adhesion and signaling events. De novo deletion of this gene was observed in a male patient with cognitive impairment, autism, and dysmorphic features^32^. Whether pirenperone can modulate the expression pattern of these genes in FXS brains remains to be determined. Especially, checking the protein expression level will provide important information on the regulatory effects of pirenperone on FMRP-dependent gene expression. In addition to the mRNA expression, the protein expression level is governed by several factors including turnover of the existing proteins, which we like to thoroughly investigate in the future studies.

The up-regulated mRNA level of the *Fmr1* gene by pirenperone implies that pirenperone may exert its effect through the modulation of synapse formation, function, and neural plasticity by adjusting the level of FMRP expression. The RNA-binding protein FMRP is reported to be involved in the translation regulation of synaptic proteins. The decrease or loss of function of FMRP will lead to behavioral deficits and decreased cognitive functions in FXS patients^33^. This will also bring about other consequences such as altered GABA receptor subunits expression^34^ as well as increased long-term depression (LTD) of excitatory synapses^35^. This leads to an imbalance in the excitatory and inhibitory signaling which is one of the common features of FXS^36,37^. This implies that the pirenperone’s up-regulation of the *Fmr1* gene leads to the increase in FMRP and might have regulated the ratio of the excitatory and inhibitory signaling thus restoring the balance and restoring some of the behavioral deficits. In this study, the most strongly affected gene is *CHL1* whose expression has elevated 4.1 times after pirenperone treatment. *CHL1* is called the cell adhesion molecule L1-like (*CHL1* or *CALL*) gene, located on chromosome 3p26.3, and it is highly expressed in the central and peripheral nervous systems. The gene guides the growth of regenerating motor axons and regulates the synaptic coverage of motor neurons^2^. Several studies have also associated the duplication as well as the deletion of the *CHL1* gene in humans with intellectual disabilities such as autism^2^. There have been recorded familial cases that presented deletion of 3p26.3 as well as the *CHL1* the gene and was suggested that a reduction of about 50% of gene expression results to cognitive deficits^2^. Our finding in conjunction with the known mechanism of *CHL1* may suggest the increase in the *CHL1* gene expression after pirenperone treatment possibly rescues FXS symptoms by modulating synaptic development or increasing the synaptic coverage of neurons.

Pirenperone inhibited head twitches induced by 5-HT treatment and amphetamine-induced hyperactivity in SD rats^2^. Many clinical studies have already mentioned that 5-HT2A receptor antagonists are useful to regulate motor symptoms of Parkinson’s disease^2^. The cortical 5-HT2A receptors can affect the function of subcortical dopamine (DA) through modulating glutamatergic cortico-striatal circuits, therefore dysregulated locomotor activity could be normalized^2^.

Regarding the 5-HT2A receptor antagonist as a target of FXS, research on the role of the modulation of 5-HT2A activity on behavioral features such as hyperactivity, memory formation, and impulsiveness can be found elsewhere. 5-HT2A receptor antagonists such as ketanserin, M100907, αMe-5HT, and MDL11939 enhanced the cognitive learning ability of Fmr1 KO and BTBR mice which are known as one of the most common ASD models^2^. Deficits in GluR1-dependent synaptic plasticity have been observed in Fmr1 KO mice^2^.

Decreased *N*-methyl-d-aspartic acid (NMDA) receptor-dependent long-term potentiation (LTP) is observed in Fmr1 KO mice because of impairments of the signal activation between Ras and PI3K/PKB that damage GluR1-dependent plasticity^2^. For the reinstatement of learning ability, synaptic GluR1 delivery is important^2^. 5-HT2A receptor regulates synaptic plasticity and glutamatergic synaptic transmission with an increase in GluR1 in pre-frontal cortex neurons and these neuronal mechanisms may underlie the modulation of long-term potentiation in Fmr1 KO mice^2^. Inhibiting 5-HT2A receptors by treatment with α-Me-5HT and MDL11939, activated Ras-PI3K/PKB signaling, GluR1-dependent synaptic plasticity, and increased the ability to learn in Fmr1 KO mice^2^.

Additionally, searching KEGG DB^38^ (https://www.genome.jp/kegg/kegg1.html), pirenperone (D05495) belongs to the chemical subclass of butyrophenone derivatives. Other components in this class include haloperidol (DG00885), melperone (DG00887), and pipamperone (DG00889) harboring dopamine D2 and 5HT2A antagonism property. Haloperidol, an antipsychotic, can be used for FXS in several respects: anxiety, agitation, and aggressive behaviors (Elizabeth Berry-Kravis’s presentation at the 13th international Fragile X conference). Since haloperidol is a dopamine D2 antagonist, it had a high affinity for D2 dopamine receptors, and moderate affinity for serotonin 5-HT2A, and 5-HT1D receptors in human brain tissue homogenates^2^. Melperone is used for the treatment of schizophrenia, sleep disorders, agitation, and mentally confused states by antagonist activity at D2 dopaminergic and 5HT2A serotonergic receptors^39^. Similarly, pipamperone can be used for the treatment of chronic psychoses and states of aggressiveness of various origins. To improve haloperidol's pharmacological effects, Janssen developed pipamperone, an agent whose pharmacological profile was distinct from haloperidol. Unlike all other known antipsychotic drugs at that time, pipamperone had significant anti-tryptamine activity^40^. Interestingly, when risperidone was created, Janssen suggested it was a more potent version of pipamperone that has a selective 5-HT2A, D1, and D4 antagonist.

Many researchers observed a low level of anxiety in Fmr1 KO mice^41^. Previous studies have reported that Fmr1 KO mice have higher rates of premature responses which means that its inhibitory controls are lacking^42^. In this study, Fmr1 KO mice revealed low anxiety, and probably high impulsivity in elevated plus maze tasks, which were rescued by pirenperone. The serotonin system has been heavily implicated in impulsivity studies in general. There are implications on the involvement of 5-HT2A polymorphisms in the G-1438A promoter^43^ regions as well as in the A-1438A allele of the 5-HT2A receptor gene^44^ as the reason behind the impulsive behavior. Furthermore, it has been reported that 5-HT2A receptor antagonism using the 5-HT2A receptor antagonist MDL100,907 attenuates impulsive behavior in mice^45,46^. As suggested by several pharmacological studies, the G-coupled 5-HT2A receptor has been reported to be responsible for impulsive activities and this means that selective 5-HT2A receptor antagonist blocks this inherent behavior^47,48^. Our current study adds to this growing data that suggests the role of the 5-HT2A antagonist in the attenuation of impulsive behavior.

Elevated blood serotonin levels or hyperserotonemia in ASD including Rett syndrome and FXS individuals have been observed in about 30% of total patients^2^. It is not clear which subtypes of serotonin receptors play important roles in the modulation of ASD phenotypes but the results from the present study suggest that 5-HT2A receptors should be given more attention.

Loss of FMRP expression leads to the hyperactivation of the extracellular-signal-regulated kinase (ERK) signaling pathways in both FXS and KO mice^2^. Stimulation of group I metabotropic glutamate receptors via 5-HT2A activates phospholipase C, splitting membrane phosphatidylinositol into inositol triphosphate (releasing intracellular Ca^2+^ from cytoplasmic stores) and diacylglycerol, a specific activator of protein kinase C (PKC). PKC activation triggers a cascade leading to MEK phosphorylation of ERK. Previous studies have reported 5-HT2A receptor function by phosphorylating ERK1 and ERK2. The 5-HT2A antagonist ketanserin inhibited the activation of the ERK signaling pathway thus activating the downstream signaling cascade^2^. Therefore, similar to kentaserin’s mechanism of action pirenperone treatment might be able to rescue the ERK hyperactivation in FXS models as well, but further studies would be necessary.

Even though there are still many questions to comprehend the mechanism of pirenperone as a potential drug candidate for FXS, we assume that the current study will provide new insights into discovering effective drug candidates for the disorder and also the possibility that 5-HT2A modulators can be drug targets for FXS.

## Materials and methods

### Animals

Fmr1 KO mice were obtained from Jackson Laboratory (Sacramento, CA, USA). Fmr1 KO mice were mated for littermates and the vaginal plug was confirmed the following day of mating. Embryo 0 (E0) was designated if the vaginal plug was checked and the day of birth was considered postnatal day 1 (P1). Dams were housed separately until their pups were weaned (P21). At P14, the genomic DNA was extracted from each mouse for the genotyping, and PCR was performed to confirm their genotype. For primary cortical neuron culture, pregnant Sprague–Dawley (SD) rats were used (Orient Bio, Seoul, Korea). Animals were maintained on a standard 12:12 h circadian cycle with consistent temperature (20–24 °C) and humidity (30–70%). All animals were able to take food and water freely.

Animal handling, caring, anesthesia, and drug administration were carried out according to the Principle of Laboratory Animal Care (NIH publication No. 85-23, revised 1985). All processes were approved by the Institutional Animal Care and Use Committee of Konkuk University (KUIACUC), Korea (KU18054).

### Materials

Poly-d-lysine (0.1 mg/ml) was obtained from Sigma (St. Louis, MO, USA) and Neurobasal medium (NBM), dimethyl sulfoxide (DMSO), 1% penicillin/streptomycin, and 1% l-glutamine were from Gibco BRL (Grand Island, NY, USA). The 2% B27 supplement was from Invitrogen (Carlsbad, CA, USA). Dulbecco's phosphate-buffered saline (DPBS) was purchased from Welgene (Gyeongsan, Korea). For quantitative real-time PCR, ABI7500 (Applied Biosystems) and SYBR® Premix Ex Taq II (2×) (RR820A, Takara, Japan) were used. For RNA extraction, the Trizol solution was obtained from Ambion (Carlsbad, CA, USA). Pirenperone (Cat No. P126, > 97% purity) was purchased from Sigma Aldrich (St. Louis, MO, USA).

### Data collection

Gene expression omnibus's (GEO) gene expression profile GSE7329 was selected to analyze DEG. The dataset contained Agilent Genome Human Microarrays data from human lymphoblastoid cells which has the following profiles; 5 autistic males with 15q11-q13 duplication, 8 autistic males with FXS, and 15 normal control males^2^. The normalized data were downloaded through the GEO database, administrated by NCBI.

### Data processing and CMap analysis

The mean gene expression difference between the FXS group (n = 11) and a healthy control group (n = 15) was derived. Two filtering criteria are sequentially applied to define DEGs for CMap input. Firstly, we select genes where their gene expression difference belongs to the top 5% (highly expressed in FXSs) or bottom 5% (lowly expressed in FXSs). Secondly, we performed a t-test for gene expression differences to select genes with a cut-off of the *p* value less than 0.05. Then, we matched the probe ID of the Agilent platform to the Affymetrix U133A probe ID. This step is necessary as CMap supports only Affymetrix probe ID for the query. Finally, we obtained 42 genes as DEGs to be used as a signature pattern for CMap analysis.

For probe ID mapping, we first map the Agilent probe ID to the official human gene symbol, then the human gene symbol is converted to Affymetrix probe ID by using the probe ID map table from Affymetrix.com. For visualization of DEGs, a Multiple Experiment Viewer (MeV) was used. Statistical analyses were performed by R-3.4.3 (https://cran.r-project.org/src/base/R-3/). For the interpretation of functional connections among selected DEGs, we used a web tool version of GeneMANIA (https://genemania.org/).

**Figure 6:**
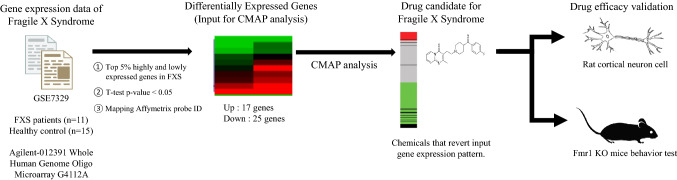
Schematic diagram of the transcriptome, CMap analysis, and therapeutic efficacy validation process.

CMap web interface provided by the Broad institute is used for our analysis (https://www.broadinstitute.org/connectivity-map-cmap). Chemicals with negative enrichment scores meeting *p* value criteria less than 0.05 were selected. As we aim to find molecules to revert disease-related gene expression signatures, chemicals with negative enrichment scores were only considered. The whole analysis scheme is explained in Fig. 6.

### Culture of rat cortical neurons

Primary cortical neurons were isolated from the embryonic day 18 (E18) brain of the SD rat as previously described^2^. Briefly, pregnant rats were anesthetized with 1% isoflurane, and the uterus was rapidly exposed to remove the fetus under sterile conditions. After the fetus was anesthetized and decapitated, the cortices were exposed and separated from brain tissue. By using mechanical trituration, cortices were divided into single cells and those cells were plated into poly-d-lysine pre-coated plates and incubated in NBM supplemented with 2% B27 supplement, 1% l-glutamine, and 1% penicillin–streptomycin in 2% CO_2_ incubator at 37 °C^2^. The density of cells was 6 × 10^5^ cells per well.

### Drug treatment (in vitro)

Pirenperone was diluted into 5, 10, and 20 μM with 0.2% DMSO and culture media for the treatment of cortical neuron cells. Treatment was conducted on the 8th day of in vitro culture of cortical neuron cells.

### RNA extraction

After 24 h of the cortical neuron cell treatment of pirenperone, total RNA was isolated using Trizol reagent. The RNA extraction protocol was followed by the supplier's instructions. After the extraction, RNA concentration was confirmed by using NanoDrop ND-1000 Spectrometer (NanoDrop Technologies Inc., Montchanin, DE, USA). cDNA synthesis was achieved after normalization of all RNA concentrations into 1 μg.

### Real-time qPCR

Quantitative real-time PCR was achieved with ABI7500 (Applied Biosystems) and results were analyzed by using the comparative threshold cycle (CT) method. Primer information is provided in Supplementary materials.

### Drug treatment (in vivo)

50 μg/kg of pirenperone was diluted in 0.2% DMSO and saline for the vehicle was given via intraperitoneal (i.p.) injection to each mouse 30 min before the behavioral experiment.

### Open field test

An exploratory hyperactivity test was performed in an open field box (40 × 40 × 30 cm). Each mouse was placed in the bottom center of an open field box and given 5 min of habituation followed by 20 min of behavior recording using EthoVision software (XT8.5)^49^. The velocity and the total distance moved were analyzed.

### Elevated plus-maze

The elevated plus-maze was performed to test the impulsivity and anxiety-like behavior^2^. The maze was composed of two open and closed arms (67 × 7 cm). The shape of the whole maze was a plus symbol, each arm facing one another and connected through a neutral square-shaped space in the center. The maze was placed 55 cm above the floor. The mouse was put on the center (neutral area) and the test started. The mouse was allowed to move across arms freely for 8 min and the movement of the mouse was recorded by using EthoVision software (XT8.5). For each arm, the total time spent and the frequency of entry were analyzed.

### Y-maze

Y-maze test was performed to assess the spatial working memory ability^2^. The maze has Y-shaped three arms (5 × 35 × 10 cm) and each corner has the same angles. Each mouse was put in the middle of the maze and was observed for their total entries and spontaneous alternations for 8 min. Spontaneous alternation was analyzed by using the following formula [total alternations/(total arm entries − 2) × 100]^2^.

### Statistical analysis

Mean ± standard error of the mean (S.E.M) was used to express data for all behavior tests and qRT-PCR analysis. Unpaired t-test and one-way ANOVA followed by Bonferroni test were used to analyze for statistical significance. Statistical significance was considered when the *p* value was less than 0.05. All statistical analyses were calculated using GraphPad Prism version 5 software (www.graphpad.com/scientific-software/prism/).

### Ethics declarations

All methods were carried out in accordance with ARRIVE guidelines. All processes were approved by the Institutional Animal Care and Use Committee of Konkuk University (KUIACUC), Korea (KU18054).

## Supplementary Information

Supplementary Tables.

## Data Availability

All data generated or analyzed during this study are included in this published article (and its supplementary information files).

## References

[1] Lozano R, Rosero CA, Hagerman RJ (2014). Fragile X spectrum disorders. Intractable Rare Dis. Res..

[2] Bear MF, Huber KM, Warren ST (2014). The mGluR theory of fragile X mental retardation. Trends Neurosci..

[3] Berry-Kravis E (2010). Seizures in fragile X syndrome: Characteristics and comorbid diagnoses. Am. J. Intellect. Dev. Disabil..

[4] Hagerman RJ (2009). Advances in the treatment of fragile X syndrome. Pediatrics.

[5] Berry-Kravis E, Sumis A, Hervey C, Mathur S (2012). Clinic-based retrospective analysis of psychopharmacology for behavior in fragile X syndrome. Int. J. Pediatr..

[6] Bagni C, Tassone F, Neri G, Hagerman R (2012). Fragile X syndrome: Causes, diagnosis, mechanisms, and therapeutics. J. Clin. Investig..

[7] Suthram S (2010). Network-based elucidation of human disease similarities reveals common functional modules enriched for pluripotent drug targets. PLoS Comput. Biol..

[8] Qu XA, Rajpal DK (2012). Applications of connectivity map in drug discovery and development. Drug Discov. Today.

[9] Ishimatsu-Tsuji Y, Soma T, Kishimoto J (2010). Identification of novel hair-growth inducers by means of connectivity mapping. FASEB J..

[10] Martinez LA, Tejada-Simon MV (2018). Pharmacological rescue of hippocampal fear learning deficits in fragile X syndrome. Mol. Neurobiol..

[11] Archer S (1990). Mode of action of the schistosomicide hycanthone: Site of DNA alkylation. Mol. Biochem. Parasitol..

[12] Hillman GR, Senft AW (1975). Anticholinergic properties of the antischistosomal drug hycanthone. Am. J. Trop. Med. Hyg..

[13] Guo J-D, Hammack SE, Hazra R, Levita L, Rainnie DG (2009). Bi-directional modulation of bed nucleus of stria terminalis neurons by 5-HT: Molecular expression and functional properties of excitatory 5-HT receptor subtypes. Neuroscience.

[14] Naghdi N, Majlessi N, Broofar F (2001). The effect of ketanserin and pirenperone injected into the CA1 region on spatial discrimination. Iran. Biomed. J..

[15] Green A, O'shaughnessy K, Hammond M, Schächter M, Grahame-Smith D (1983). Inhibition of 5-hydroxytryptamine-mediated behaviour by the putative 5-HT2 antagonist pirenperone. Neuropharmacology.

[16] Nichols DE (2016). Psychedelics. Pharmacol. Rev..

[17] Powell SK, Kleinman HK (1997). Neuronal laminins and their cellular receptors. Int. J. Biochem. Cell Biol..

[18] Wei M-H (1998). In silico-initiated cloning and molecular characterization of a novel human member of the L1 gene family of neural cell adhesion molecules. Hum. Genet..

[19] Palumbo O (2015). De novo microduplication of CHL1 in a patient with non-syndromic developmental phenotypes. Mol. Cytogenet..

[20] Li C, Liu C, Zhou B, Hu C, Xu X (2016). Novel microduplication of CHL1 gene in a patient with autism spectrum disorder: A case report and a brief literature review. Mol. Cytogenet..

[21] Salyakina D (2011). Copy number variants in extended autism spectrum disorder families reveal candidates potentially involved in autism risk. PLoS ONE.

[22] Hu J (2015). CNTN6 copy number variations in 14 patients: A possible candidate gene for neurodevelopmental and neuropsychiatric disorders. J. Neurodev. Disord..

[23] Iqbal Z (2013). Homozygous and heterozygous disruptions of ANK3: At the crossroads of neurodevelopmental and psychiatric disorders. Hum. Mol. Genet..

[24] Bi C (2012). Mutations of ANK3 identified by exome sequencing are associated with autism susceptibility. Hum. Mutat..

[25] Wang Y, Südhof TC (2003). Genomic definition of RIM proteins: Evolutionary amplification of a family of synaptic regulatory proteins. Genomics.

[26] Weidenhofer J, Scott RJ, Tooney PA (2009). Investigation of the expression of genes affecting cytomatrix active zone function in the amygdala in schizophrenia: Effects of antipsychotic drugs. J. Psychiatr. Res..

[27] Connor R, Key B (2002). Expression and role of Roundabout-1 in embryonic *Xenopus* forebrain. Dev. Dyn. Off. Publ. Am. Assoc. Anat..

[28] Lee JS, Ray R, Chien CB (2001). Cloning and expression of three zebrafish roundabout homologs suggest roles in axon guidance and cell migration. Dev. Dyn. Off. Publ. Am. Assoc. Anat..

[29] Hoischen A (2010). De novo mutations of SETBP1 cause Schinzel-Giedion syndrome. Nat. Genet..

[30] Laborde R (2013). SETBP1 mutations in 415 patients with primary myelofibrosis or chronic myelomonocytic leukemia: Independent prognostic impact in CMML. Leukemia.

[31] O’Roak BJ (2012). Sporadic autism exomes reveal a highly interconnected protein network of de novo mutations. Nature.

[32] Stark Z, Bruno DL, Mountford H, Lockhart PJ, Amor DJ (2010). De novo 325 kb microdeletion in chromosome band 10q25. 3 including ATRNL1 in a boy with cognitive impairment, autism and dysmorphic features. Eur. J. Med. Genet..

[33] Darnell JC (2011). FMRP stalls ribosomal translocation on mRNAs linked to synaptic function and autism. Cell.

[34] Fatemi SH, Folsom TD (2015). GABA receptor subunit distribution and FMRP–mGluR5 signaling abnormalities in the cerebellum of subjects with schizophrenia, mood disorders, and autism. Schizophr. Res..

[35] Bear MF, Huber KM, Warren ST (2004). The mGluR theory of fragile X mental retardation. Trends Neurosci..

[36] Martin BS, Corbin JG, Huntsman MM (2014). Deficient tonic GABAergic conductance and synaptic balance in the fragile X syndrome amygdala. J. Neurophysiol..

[37] Gatto CL, Broadie K (2010). Genetic controls balancing excitatory and inhibitory synaptogenesis in neurodevelopmental disorder models. Front. Synaptic Neurosci..

[38] Kanehisa M, Goto S (2000). KEGG: Kyoto encyclopedia of genes and genomes. Nucleic Acids Res..

[39] Whiskey E, Vavrova M, Gaughran F, Taylor D (2011). Melperone in treatment-refractory schizophrenia: A case series. Ther. Adv. Psychopharmacol..

[40] López-Muñoz, F. & Álamo González, C. The pharmacological role and clinical applications of antipsychotics’ active metabolites: Paliperidone versus risperidone (2013).

[41] Eadie BD (2009). Fmr1 knockout mice show reduced anxiety and alterations in neurogenesis that are specific to the ventral dentate gyrus. Neurobiol. Dis..

[42] Moon J-S (2006). Attentional dysfunction, impulsivity, and resistance to change in a mouse model of fragile X syndrome. Behav. Neurosci..

[43] Preuss UW, Koller G, Bondy B, Bahlmann M, Soyka M (2001). Impulsive traits and 5-HT2A receptor promoter polymorphism in alcohol dependents: Possible association but no influence of personality disorders. Neuropsychobiology.

[44] Nomura M, Nomura Y (2006). Psychological, neuroimaging, and biochemical studies on functional association between impulsive behavior and the 5-HT2A receptor gene polymorphism in humans. Ann. N. Y. Acad. Sci..

[45] Higgins GA, Enderlin M, Haman M, Fletcher PJ (2003). The 5-HT2A receptor antagonist M100, 907 attenuates motor and'impulsive-type'behaviours produced by NMDA receptor antagonism. Psychopharmacology.

[46] Winstanley CA, Theobald DE, Dalley JW, Glennon JC, Robbins TW (2004). 5-HT2A and 5-HT2C receptor antagonists have opposing effects on a measure of impulsivity: Interactions with global 5-HT depletion. Psychopharmacology.

[47] Fink LH (2015). Individual differences in impulsive action reflect variation in the cortical serotonin 5-HT2A receptor system. Neuropsychopharmacology.

[48] Anastasio NC (2011). The serotonin (5-Ht) 5-Ht2a receptor: Association with inherent and cocaine-evoked behavioral disinhibition in rats. Behav. Pharmacol..

[49] Adil KJ (2022). Behavioral deficits in adolescent mice after sub-chronic administration of NMDA during early stage of postnatal development. Biomol. Ther..

